# Evaluation of a mobile health approach to improve the Early Warning System of influenza surveillance in Cameroon

**DOI:** 10.1111/irv.12747

**Published:** 2020-05-14

**Authors:** Chavely Gwladys Monamele, Loique Landry Messanga Essengue, Mohamadou Ripa Njankouo, Hermann Landry Munshili Njifon, Jules Tchatchueng, Mathurin Cyrille Tejiokem, Richard Njouom

**Affiliations:** ^1^ Laboratory of Virology Centre Pasteur of Cameroon Yaoundé Cameroon; ^2^ Laboratory of Epidemiology Centre Pasteur of Cameroon Yaoundé Cameroon; ^3^ Centre Pasteur of Cameroon Annex of Garoua Garoua Cameroon

**Keywords:** Cameroon, data collection, Early Warning System, influenza, paper‐based system, short message service

## Abstract

**Background:**

Rapid reporting of surveillance data is essential to better inform national prevention and control strategies.

**Objectives:**

We compare the newly implemented smartphone‐based system to the former paper‐based and short message service (SMS) for collecting influenza epidemiological data in Cameroon.

**Methods:**

Of the 13 sites which collect data from persons with influenza‐like illness (ILI), six sites send data through the EWS, while seven sites make use of the paper‐based system and SMS. We used four criteria for the comparison of the data collection tools: completeness, timeliness, conformity and cost.

**Results:**

Regarding the different collection tools, data sent by the EWS were significantly more complete (97.6% vs 81.6% vs 44.8%), prompt (74.4% vs n/a vs 60.7%) and of better quality (93.7% vs 76.1% vs 84.0%) than data sent by the paper‐based system and SMS, respectively. The average cost of sending a datum by a sentinel site per week was higher for the forms (5.0 USD) than for the EWS (0.9 USD) and SMS (0.1 USD). The number of outpatient visits and subsequently all surveillance data decreased across the years 2017‐2019 together with the influenza positivity rate from 30.7% to 28.3%. Contrarily, the proportion of influenza‐associated ILI to outpatient load was highest in the year 2019 (0.37 per 100 persons vs 0.28 and 0.26 in the other 2 years).

**Conclusion:**

All sentinel sites and even other disease surveillance systems are expected to use this tool in the near term future due to its satisfactory performance and cost.

## 1 INTRODUCTION

In recent years, influenza surveillance that was essentially virological expanded to include more epidemiological information to complement the virological data collected by the Global Influenza Surveillance and Response System (GISRS).^1^ The 2009 influenza pandemics highlighted the need for rapid reporting of cases to assess the severity of the disease, define risk factors for severe outcome and to better inform national prevention and control strategies. This has urged many countries to establish surveillance systems for the early detection of public health emergencies and detection of potential pandemic influenza strains.^2^

Reporting of surveillance data has mostly made use of paper‐based systems, mobile phone–based systems and Web‐based systems. Among these, mobile and Internet technologies have been successfully used for EWS in several countries and settings.^2^, ^3^, ^4^, ^5^ In Cameroon, there has been progress in the collection tools for influenza epidemiological data from forms to SMS (short message service) to smartphone using the Internet in order to improve on the timeliness of data collected. The implementation of the EWS, a Web‐based system that makes use of smartphones, within the influenza surveillance in 2017 started with a few sentinel sites in Cameroon for more real‐time analyses of data collected and in the preparedness of a future pandemic event.

We evaluate here the performance of the EWS as compared to prior tools for collecting influenza epidemiological data and estimate the annual proportion of influenza‐associated illness among total outpatient visits in Cameroon.

## 2 METHODS

### 2.1 Description of the influenza surveillance system

For more than a decade, the Centre Pasteur of Cameroon has been designated the National Influenza Centre of Cameroon by the Ministry of Health and by the World Health Organization. In 2019, the influenza surveillance system comprised 16 sites distributed in 7 of the 10 administrative regions of the country. Among these, 13 sites collect data from outpatients, while 3 sites collect data from hospitalized patients with a severe acute respiratory infection (SARI). This surveillance system generates two main types of data: epidemiological data from sentinel sites and virological data from laboratory analysis of samples collected. Epidemiological data are collected weekly from sentinel sites and comprise information on the number of consultations, number of febrile illness, number of acute respiratory infections (ARI), number of influenza‐like illness (ILI) and number of samples collected. Meanwhile, virological data obtained mostly comprise the influenza status of each individual sample collected. Nasopharyngeal and/or oropharyngeal swabs collected from the sites are analysed for the presence of influenza virus using the gold standard assay, rRT‐PCR, as previously described.^6^

### 2.2 Evolution in the tools for collecting epidemiological data

Tools for the collection of epidemiological data from sentinel sites have gradually evolved over the years from forms (paper‐based system) to SMS to the smartphones (EWS). Initially, all epidemiological data were sent through the paper‐based system together with the respiratory samples. However, some major issues encountered with this system were the lack of complete data and timeliness. In September 2012, weekly reporting by SMS started at the sentinel sites in addition to the paper‐based system. Data sent by SMS comprised reduced information as compared to the forms, with two parameters reported by age groups, that is number of consultations and number of ILI. This reduced reporting via SMS was implemented to enable timely reporting of the minimum essential data in the WHO FluID platform (https://extranet.who.int/fluid/Login.aspx?ReturnUrl=%2ffluid%2f) since sentinel sites located in distant regions had difficulties sending the forms on time. Data sent by SMS could be received by one of the two telephone devices located at the NIC. Once the form or SMS data are received, they are entered manually in an Excel database.

Recently, reporting via the EWS with smartphones was initiated in a few sentinel sites in order to improve still on the timeliness of data received. The EWS makes use of *Event Capture*, an Android application which enables to capture and submit events (https://play.google.com/store/apps/details?id=org.hisp.dhis.android.eventcapture&hl=en). This system first started in January 2017 with sites located in the same town as the NIC (Yaounde) for a better coordination of this novel tool, and then was extended to sites located in the Northern region of Cameroon (Garoua) in August 2018. The EWS started with weekly reporting, but changed during the second phase of implementation to daily reporting for a better preparedness to a future pandemic event or in case of any unusual rise in influenza activity. Daily data sent through the EWS are aggregated into weekly data and extracted automatically in the server at the NIC.

Of the 13 sites which collect data from persons with ILI, 6 sites send data through the EWS, while the remaining 7 sites make use of forms and SMS. Of the 6 sites supposed to send data through the EWS, one had not sent any data and was discarded in the analysis.

### 2.3 Method of comparison of collection tools

We used four criteria for the comparison of the epidemiological data collection tools: completeness, timeliness, conformity and cost. Proportions of each criterion were compared among all three tools. Completeness refers to data of the 52 epidemiological weeks that was successfully sent. For the EWS, completeness also involved sending all five or six daily data corresponding to the working days of the week.

$$\text{Completeness}\;\left(\%\right)=\text{Number of data received}/\text{Number of data expected}\times 100$$

Timeliness refers to data that were sent timely, that is within three days following the end of the reporting period.

$$\text{Timeliness}\;\left(\%\right)=\text{Number of data received on time}/\text{Number of data received}\times 100$$

Conformity refers to data that had no errors. We considered here as errors data with totals of each parameter wrongly calculated, incoherence of data (number of ILI > number of ARI OR number of febrile illness > number of consultations), errors in selecting the epidemiological week and presence of missing values in data sent.

$$\text{Conformity}\;\left(\%\right)=\text{Number of quality data received}/\text{Number of data received}\times 100$$

Cost corresponds to the average cost in USD of sending one datum by a sentinel site per week. The cost of sending one datum through the EWS comprised the weekly cost of Internet provision necessary to send the data. The cost of sending one datum through the SMS comprised the cost of the SMS in accordance with the network provisioner. The cost of sending data through the paper‐based system comprised the transport cost for sending the notification forms alongside the collected samples. We exclusively use 2019 data for comparisons among the different tools to ease analyses and to minimize bias.

### 2.4 Statistical analysis

Comparison of proportions of the different collection tools was performed using the chi‐square test in IBM SPSS statistical software version 22.0 and considering the proportions obtained with the EWS as reference values. Meanwhile, the Student *t* test was used to compare means. The annual proportional contribution of influenza‐associated ILI to outpatient load (*P*) was calculated using the method described in WHO's Manual for Estimating Disease Burden Associated with Influenza.^7^ The burden of influenza‐associated ILI to annual outpatient load was calculated by estimating the proportion of the total number of influenza‐associated ILI visits among all outpatient visits. For more adequate analyses, virological data were considered for the sites that had consistently collected at least 75% of complete epidemiological data during the years 2017‐2019.

$$P\;\left(\%\right)=\text{Number of influenza}\;-\;\text{associated ILI visits}/\text{Total number of outpatient visits at the sentinel site}\times 100$$

## 3 RESULTS

### 3.1 Description of epidemiological data collected from 2017‐2019

The number of outpatient visits and subsequently all surveillance data decreased across the years 2017‐2019. The proportion of febrile illness, ARI and ILI with respect to the number of consultations was highest in the 1‐4 years age group in all 3 years, whereas the lowest proportions of the three epidemiological data were observed in the ≥ 50 years age group (Table 1).

**TABLE 1:** Epidemiological data collected with respect to virological data

Age group	Consultation^a^	Febrile illness N (%)	ARI N (%)	ILI^b^ N (%)	No. tested	Influenza positive N (%)^c^	Inf.‐associated ILI cases^d^	Inf.‐associated ILI to outpatient load per 100 persons (%)^e^
2017								
<1	18 196	4917 (27.0)	836 (4.6)	303 (1.7)	286	47 (16.4)	50	0.27
1‐4	20 715	6117 (29.5)	1006 (4.9)	547 (2.6)	518	185 (35.7)	195	0.94
5‐14	17 246	4918 (28.5)	435 (2.5)	164 (1.0)	155	67 (43.2)	71	0.41
15‐49	65 908	6984 (10.6)	702 (1.1)	249 (0.4)	189	52 (27.5)	69	0.10
≥50	24 824	1793 (7.2)	258 (1.0)	81 (0.3)	62	14 (22.6)	18	0.07
Unknown	0	0	0	0	86	33 (38.4)	/	/
Total	146 889	24 729 (16.8)	3237 (2.2)	1344 (0.9)	1296	398 (30.7)	413	0.28
2018								
<1	18 046	4636 (25.7)	924 (5.1)	260 (1.4)	200	50 (25.0)	65	0.36
1‐4	18 542	5953 (32.1)	1173 (6.3)	491 (2.6)	354	109 (30.8)	151	0.82
5‐14	16 079	5073 (31.6)	520 (3.2)	177 (1.1)	138	45 (32.6)	58	0.36
15‐49	66 579	8251 (12.4)	967 (1.5)	229 (0.3)	162	57 (35.2)	81	0.12
≥50	20 952	2072 (9.9)	311 (1.5)	77 (0.4)	48	15 (31.3)	24	0.11
Unknown	0	0	0	0	26	2 (7.7)	/	/
Total	140 198	25 985 (18.5)	3895 (2.8)	1234 (0.9)	928	278 (30.0)	370	0.26
2019								
<1	12 452	3206 (25.7)	771 (6.2)	298 (2.4)	158	32 (20.3)	60	0.48
1‐4	12 433	4122 (33.2)	862 (6.9)	421 (3.4)	240	73 (30.4)	128	1.03
5‐14	10 423	3004 (28.8)	488 (4.7)	235 (2.3)	102	42 (41.2)	97	0.93
15‐49	45 307	6024 (13.3)	806 (1.8)	231 (0.5)	105	33 (31.4)	73	0.16
≥50	14 227	1476 (10.4)	198 (1.4)	54 (0.4)	37	9 (24.3)	13	0.09
Unknown	0	0	0	0	140	32 (22.9)	/	/
Total	94 842	17 832 (18.8)	3125 (3.3)	1239 (1.3)	782	221 (28.3)	351	0.37

Note

d = (b) × (c); e = (d)/(a) × 100.

Influenza positivity rate decreased across the years from 30.7% to 28.3% with predominant age group varying from one year to another. Contrarily, the proportion of influenza‐associated ILI to outpatient load was highest in the year 2019 (0.37 per 100 persons vs 0.28 and 0.26 in the other 2 years). In all 3 years, the proportion of influenza‐associated ILI to outpatient load was highest in the 1‐4 years age group and lowest in the ≥ 50 years age group.

### 3.2 Trends in epidemiological and virological surveillance data

**FIGURE 1:**
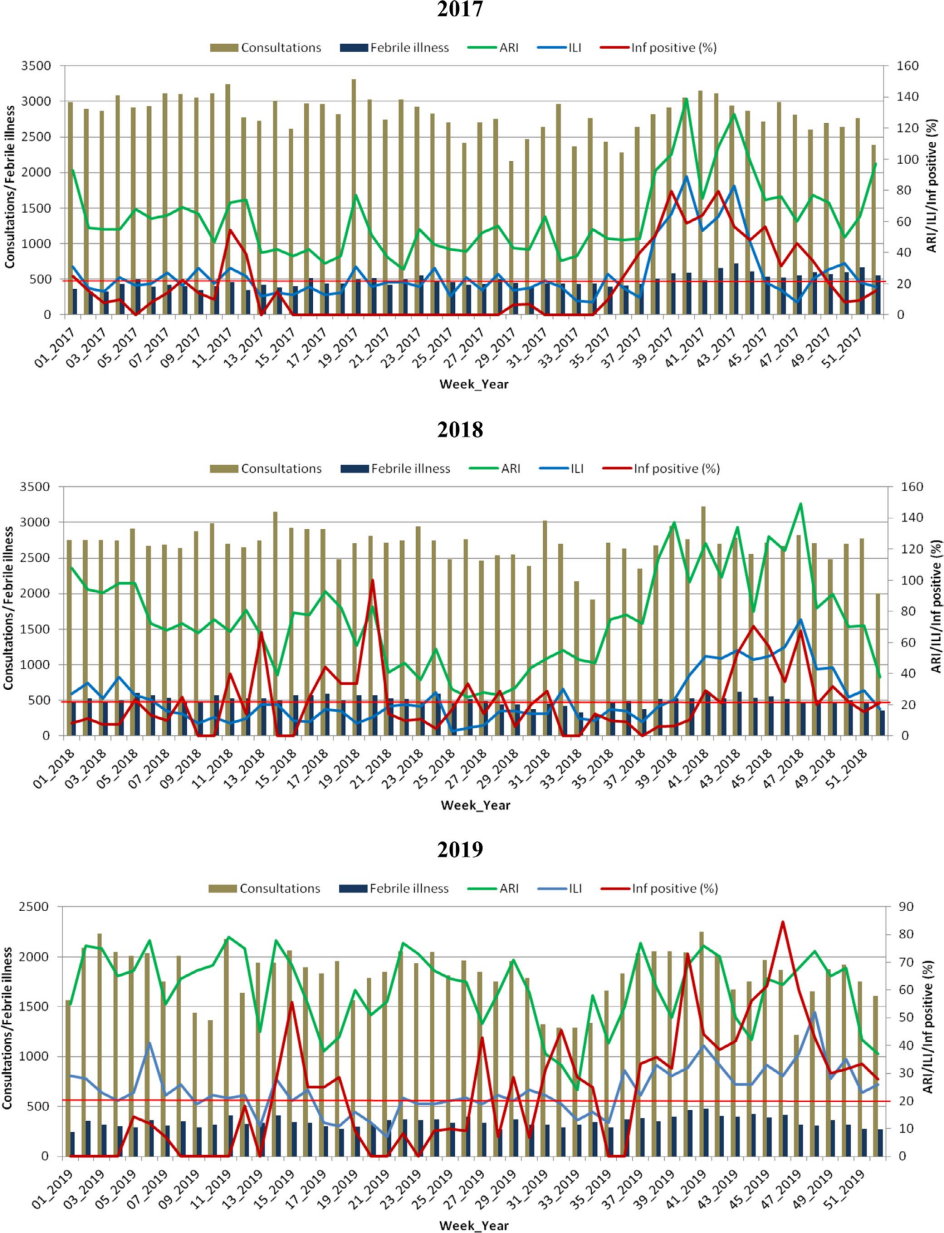
Epidemiological trends and weekly distribution of influenza virus

Figure 1 shows the epidemiological trends and weekly distribution of influenza virus during the years 2017‐2019. Globally, we noted some visual correlation between influenza positivity rate and the epidemiological data collected. In 2017, influenza positivity rate correlated with number of ARI and ILI. Meanwhile, in 2018‐2019, influenza positivity rate correlated with numbers of ARI and ILI between week 37 and week 52. Moreover, periods with consistently high ILI levels (>20) were associated with increased influenza activity. Higher influenza activity was observed at the end of the year between week 37 and week 52. Meanwhile, a small peak in influenza activity between week 11 and week 21 did not correlate with any epidemiological data.

### 3.3 Comparison of the tools for collecting surveillance data

Concerning the tools used in collecting epidemiological data; of the 364 data that were expected to be sent by forms, 81.6% were eventually sent to the NIC, 76.1% of which were conform. Meanwhile, data sent by SMS were 44.8% complete, 60.7% prompt and 84.0% conform. Data sent via the EWS on the other hand was complete at 97.6%, with a timeliness of 74.4% and conformity of 89.5%. Completeness of daily data collected via the EWS was moderate at 77.3% (Table 2). Regarding the reasons for non‐conformity of data reported; errors in the forms and SMS were mostly due to calculation of the totals of each parameter (32.9% vs 71.4%), incoherence of data (64.5% vs 10.7%) and errors in selecting the epidemiological week (2.6% vs 17.9%). Non‐conformity observed with the EWS was essentially due to missing values in data sent. The average cost of sending a datum by a sentinel site per week was higher for the forms (5.0 USD) than for the EWS (0.9 USD) and SMS (0.1 USD).

**TABLE 2:** Comparison of completeness, timeliness and conformity of collection tools

	EWS (Ref) N = 260	Forms N = 364		SMS N = 364	
		N (%)	*P*‐value	N (%)	*P*‐value
Completeness (%)	254 (97.6)/201 (77.3)^a^	297 (81.6)	<.001	163 (44.8)	<.001
Timeliness (%)	192 (74.4)	n/a		99 (60.7)	.001
Conformity	238 (93.7)	226 (76.1)	<.001	137 (84.0)	.025
Average cost/week (USD)	0.9	5.0	<.001	0.1	<.001

Note

N = expected data. n/a: not applicable; *P*‐values are related to comparison of proportions or average with respect to the EWS considered here as reference.

^a^ Completeness related to sending the 5 or 6 daily data via the EWS.

**FIGURE 2:**
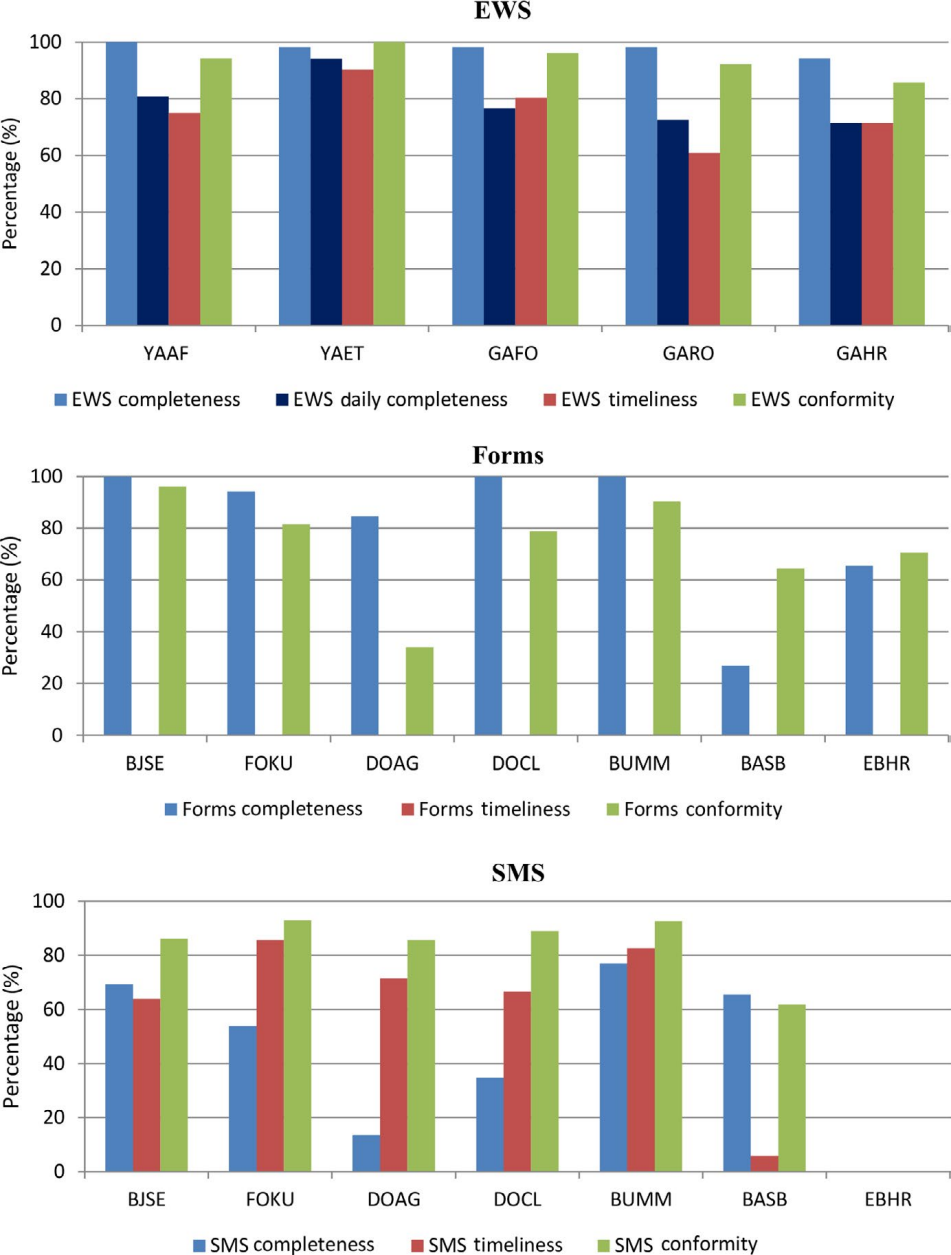
Sentinel site performance based on the different data collection tools. Sentinel sites are denoted by four‐letter codes; YAAF = CMS Ambassade de France (Yaounde); YAET = CSI d'Etoudi (Yaounde); GAFO = Hôpital de Foulbere (Garoua); GARO = CSI de Roumde Adjia (Garoua); GAHR = Garoua Regional Hospital (Garoua); BJSE = CSI de Bandjoun (Bandjoun); FOKU = CSI de Kueka (Foumban); DOAG = Hôpital Albert le Grand (Douala); DOCL = Hôpital Catholique de Log Pom (Douala); BUMM = Mount Mary Hospital (Buea); BASB = Polyclinic St Blaise (Bamenda); EBHR = Ebolowa Regional Hospital

Regarding the performance of the collection tools by sentinel sites, the majority of data sent by the EWS were ≥94% complete (weekly data), 70%‐94% had complete daily data, 70%‐90% were sent on time, and 86%‐100% were of good quality. Data sent by forms were 85%‐100% complete for the majority; meanwhile, two sites (BASB and EBHR) had 27% and 65% complete data. Conformity of data was 71%‐96% for five of the seven sites; meanwhile, DOAG and BASB had 34% and 64% conformity, respectively. Data sent by SMS were 35%‐77% complete for most sites; meanwhile, one site (DOAG) had 13% complete data. Timeliness of data sent by SMS was 64%‐86% for five sites with one site having 6% timeliness (BASB). SMS conformity on the other hand was 86%‐93% for most sites and 62% for BASB. One of the sentinel sites sent no SMS data. Figure 2 shows the performance of each sentinel site based on the three data collection tools.

## 4 DISCUSSION

This study aimed to compare the performance of the EWS to the paper‐based system and to the SMS in reporting influenza epidemiological data with respect to four selected criteria. Results showed that the EWS had significantly better performance in sending complete, prompt and conform data at a low cost.

Regarding completeness of data, SMS had the lowest proportion of complete data. This can be attributed in part to the disruption of the mobile network for over 3 months in the main telephone device through which the SMS should be sent. Also, some focal points raised the work overload as a reason for not sending SMS data and preferred making snapshots of the epidemiological forms which they consider easier to send via mobile applications (WhatsApp). Another reason for the low proportion of complete data received is the security issue faced by two regions in which the sentinel sites are located. Focal points in these regions (BASB and BUMM) reported facing difficulties conducting their daily activity including the surveillance activity. Meanwhile, not all forms containing epidemiological data were sent to the NIC. The main reason for this is the small number of persons involved in the influenza surveillance activity at sentinel sites and the high workload. As reported by the influenza surveillance team in Zambia, having a dedicated surveillance staff may increase enrolment rates. However, hiring new staff would decrease the sustainability of the surveillance system.^8^

Routinely, timeliness of the paper‐based system in Cameroon is generally low and is not evaluated due to the fact that sentinel sites located in further regions do not send data when there are no samples accompanying it. Meanwhile, timeliness of SMS data was lower than the EWS although both had moderately good values. The workload has been reported by the focal points as the main reason for not sending timely data. Timeliness of the SMS was lower than reported by other influenza surveillance systems in Africa^2^, ^9^ but higher than that observed in 2014‐2015 with the IDSR in Madagascar.^10^

Regarding data quality, there were fewer errors in data sent through the EWS than data sent by forms or SMS. This is not surprising since the most commonly noted sources of error with the forms and SMS were corrected during the implementation and programming of the EWS. However, some data presented with missing information in the EWS, and this was corrected automatically in the system once the error was identified. A similar study in Kenya reported as well less errors in smartphones compared to the paper‐based questionnaire.^11^ Generating automated weekly bulletins for reporting performance, trends and summary of data collected by each site could help identify erroneous data rapidly, improve on site performance and help in driving public health actions as noted by other EWS.^3^

The average cost of sending a datum by a sentinel site per week was lower for the SMS (0.1 USD) than for the forms (5 USD) and EWS (0.9 USD). However, SMS data still need to be entered manually in the database and this could be a potential source of error. The cost of sending data by the paper‐based system was high because the forms are generally sent together with the samples. Meanwhile, the annual average cost for sending data through the EWS did not take into consideration the cost of setting up the electronic data collection system which is greater due to the high cost of electronic equipment and operating software. However, once these initial expenses have been handled, the EWS remains more cost‐effective than using the paper‐based system and SMS especially considering the possibility of analysing the data on real time. Similar findings were reported in Kenya where the EWS was found to be more cost‐effective than the paper‐based system.^11^

The estimated incidence of influenza‐associated ILI outpatient visits in 2019 (0.37) was lower than that observed in Senegal within the cumulative period of 2013‐2015 (0.9/100 population), in the USA (8.7/1000 population) and in Thailand (14.2/1000 population).^4^, ^12^, ^13^ Our results might underestimate the burden of influenza‐associated ILI in Cameroon since the majority of patients with ILI do not refer to any health facility for treatment. Also, a hospital admission survey is essential in order to have more accurate burden of disease estimate using the catchment population.^7^ Nevertheless, the 1‐4 and 5‐14 years age groups had higher proportions of influenza‐associated ILI outpatient visits (1.03 and 0.93) confirming that there are risk groups on which targeted prevention strategies should be addressed. A previous study from Cameroon has indeed confirmed higher transmission rates of influenza virus in this age groups probably due to high contact rates in schools.^6^ There was one peak of influenza activity in 2019 between week 39 and week 52 and this was slightly correlated with ILI levels. This could be used in setting up the alert thresholds in the EWS. This result corroborates with previous findings which showed that the major period for influenza activity in Cameroon is between the months of September to December.^6^ Although ILI and ILI% are better indicators for use in EWS, as they are easily generated, these indicators may results in bias since illnesses other than influenza may present with ILI.^14^, ^15^

## 5 CONCLUSION

At the end of this study, which aimed to evaluate the performance of the EWS in collecting epidemiological data as compared to the paper‐based system and the SMS, we found that the EWS had significantly satisfactory performance based on the four selected criteria for evaluation. Also, after implementation, considering the low cost of approximately 0.9 USD for sending one complete surveillance data per site, this tool could be proposed for national surveillance systems. All sentinel sites and even other disease surveillance systems are expected to use this tool in the near term future due to its satisfactory performance and cost. The next step in the EWS is to integrate alert threshold for influenza virus circulation in Cameroon based on previous surveillance data.

## CONFLICT OF INTEREST

The authors declare that they have no competing interests.

## AUTHOR CONTRIBUTIONS

**Chavely Gwladys Monamele: **Formal analysis (lead); methodology (lead); writing‐original draft (lead); writing‐review & editing (equal). **Loique Landry Messanga Essengue: **Data curation (equal); formal analysis (supporting); software (equal); writing‐review & editing (equal). **Mohamadou Ripa Njankouo: **Investigation (equal); writing‐review & editing (equal). **Hermann Landry Munshili Njifon: **Investigation (equal); writing‐review & editing (equal); **Jules Tchatchueng: **Data curation (equal); software (equal); writing‐review & editing (equal); **Mathurin Cyrille Tejiokem: **Conceptualization (supporting); methodology (supporting); supervision (equal); writing‐review & editing (equal). **Richard Njouom: **Conceptualization (lead); funding acquisition (lead); methodology (supporting); supervision (equal); validation (lead); writing‐review & editing (equal).
